# Oral Microbiota, Bacterial Infections, Antibiotic Prescriptions, and Antimicrobial Resistance in Children

**DOI:** 10.3390/microorganisms11081927

**Published:** 2023-07-28

**Authors:** Alessandra Amato

**Affiliations:** Department of Neuroscience, Reproductive Science and Dentistry, University of Naples Federico II, 80131 Naples, Italy; aale.amato@gmail.com

The oral cavity hosts the second most diverse microbial community, over 700 bacterial taxa, with the human gut having the widest diversity. The oral cavity provides numerous surfaces for biofilm proliferation, including the keratinized gingiva, buccal mucosa, tongue, teeth, hard palate, palatal folds, and tonsillar crypts. The oral microbiome of children is shaped by early life factors such as host genetics, mode of birth, and mode of nutrition (breastfeeding or artificial feeding). The creating of the oral microbiome begins during fetal development and becomes increasingly complicated after birth. The first few years of life are crucial for diversification, with the oral biofilm assembling and reaching over 32 species-level taxa by about two years of age [1,2].

Children are susceptible to a number of bacterial infections in the orofacial region [3], and these infections are similar in most cases to those experienced by adults [4]. The primary cause of orofacial infections is related to dental problems, with dental caries and their associated conditions, such as pulpitis, pulp necrosis, apical periodontitis, and periapical abscess, being the most prevalent [5].

Childhood caries should be promptly treated as they might be associated with pain, dental space loss, failure to thrive, and disruption to quality of life [5,6]. Delay in the treatment of dental decay allows the infection to progress, causing infection of the tooth pulp (pulpitis), which is associated with a more severe type of pain than dental caries [6]. Untreated pulpitis may progress to pulpal necrosis, periapical abscess, or dentoalveolar abscess, the latter being localized in the gingiva of the affected tooth [6]. The periapical abscess may sometimes drain in the sulcus by forming a sinus tract, clinically manifesting as a parulis [3]. Another consequence of periapical infection is the formation of a pulp polyp, a chronic hyperplastic lesion forming in the pulp chamber due to long-standing infection [3]. The sinus tract, parulis, and pulp polyp experience localized inflammatory lesions, which are treated with operative intervention and not with antibiotic therapy [7].

Moreover, sepsis is a serious complication of the carious primary teeth, which can progress to cellulitis; accordingly, a substantial proportion of facial cellulitis is attributed to odontogenic infections [6]. If not properly treated, cellulitis can spread to the floor of the mouth, leading to Ludwig’s angina, compromising the airway, or it can lead to blindness and involvement of the mediastinum and spinal column [8]. Therefore, early treatment of odontogenic infections can prevent these morbidities; however, antibiotic administration may be needed when the infection becomes severe [9]. 

Children are also susceptible to periodontal diseases, which can be classified into several categories [10]. The most common are biofilm-related diseases, which are secondary to poor biofilm control and can be treated with operative intervention through scaling and oral hygiene measures [11]. 

Gingivitis, characterized by reversible bleeding from the gingiva with no loss of periodontal attachment, is highly prevalent in children and adolescents [5]. Periodontitis involves the irreversible loss of attachment and can occur in childhood or adolescence [6]. Although rare in adolescence, chronic periodontitis is generally mechanically managed without using antimicrobials or antibiotics [12]. Conversely, aggressive periodontitis and necrotizing periodontal disease benefit from adjunctive antibiotic therapy [13]. Aggressive periodontitis is multifactorial and characterized by a relatively rapid course of tissue destruction [13]. As such, timely intervention is crucial [14]. In these cases, systemic antibiotics have yielded substantial benefits [13,15]. The antibiotics most commonly used for aggressive periodontitis include tetracyclines, amoxicillin, metronidazole, macrolides, clindamycin, and ciprofloxacin [3,7,13,15].

Necrotizing periodontal diseases include necrotizing ulcerative gingivitis, periodontitis, and stomatitis [16]. They share common clinical features consisting of an acute inflammatory process and the presence of periodontal destruction [6]. These diseases are more prevalent in developing countries, particularly those in sub-Saharan Africa, and predominantly affect young children with pre-existing debilitating conditions [17].

The antibiotics most commonly used in odontogenic and periodontal infections include β-lactams, macrolides, lincosamides, nitroimidazoles, and tetracyclines, which are best suited for dental problems [3,7,13,15].

β-lactam antibiotics, which include penicillins, cephalosporins, monobactams, and carbapenems, have a bactericidal effect, meaning they destroy bacteria. The molecular targets of this class of antibiotics are penicillin-binding proteins (PBPs), which are enzymes that catalyze the reactions (such as transpeptidases and carboxypeptidases) essential for the synthesis of peptidoglycan [18]. β-lactam antibiotics can be inactivated by bacterial β-lactamases, leading to the development of antimicrobial resistance. For this reason, β-lactam antibiotics are often combined with β-lactamase inhibitors such as clavulanic acid, sulbactam, and tazobactam [18,19].

The macrolides, including erythromycin, clarithromycin, and azithromycin, are antibiotics with bacteriostatic activity that block or limit bacterial replication. However, at high concentrations, they can have a bactericidal effect. They inhibit protein synthesis by binding to the 50S subunit of the bacterial ribosome [20].

Tetracyclines inhibit protein synthesis by binding to the 30S ribosomal subunit, preventing aminoacyl-tRNA access to the acceptor site of the ribosomal complex. Tetracyclines can enter the bacterial cell by crossing the outer cell membrane through porins. Instead of assuming a lipophilic, dissociated form, they can pass through the cytoplasmic membrane [20].

Lincosamides, such as clindamycin, block protein synthesis at various stages by binding to the 50S ribosomal subunit. They bind to a site close to the macrolide binding site, so the possibility exists that the two antibiotics may interfere with each other [21].

Metronidazole, from the nitroimidazole class, produces a cytotoxic effect by interacting with the DNA of anaerobic bacteria [22]. Its cytotoxicity is achieved through the nitro group present in the molecule, which, once in the molecule, is reduced and thus acts only on anaerobic bacteria [13,22]. 

Such antibiotics are generally prescribed for the treatment of odontogenic and nonodontogenic acute and chronic infections and the prophylaxis of focal infections in high-risk patients (those suffering from systemic diseases such as endocarditis or congenital heart disease) and of focal and systemic infections in patients requiring dental treatment or oral surgery [23]. Nevertheless, antibiotics are widely prescribed in dental practice for children, with estimates suggesting that 66.4% of dental prescriptions in England are antibacterial drugs [24]. This has led to the overuse and misuse of antibiotics for nonindicated clinical conditions, such as pain relief, irreversible pulpitis, and localized dentoalveolar abscess, as well as prescribing broad-spectrum antibiotics for infections that can be treated with narrow-spectrum antibiotics, prescribing antibiotics for long periods, and adopting inappropriate dosing regimens, exposing pediatric dental patients to a multitude of potential adverse reactions [9,25]. 

Early life antibiotic exposure is thought to change the intestinal microbiota, having subsequent adverse and long-term effects such as obesity [26]. Generally, children treated with antibiotics are at risk of developing allergies and asthma [27]. Other complications associated with antibiotic prescribing in pediatric populations include superinfections with Candida species, photosensitivity, gastrointestinal disturbances, and developmental enamel defects on the permanent first molars and maxillary central incisors [25,27,28]. Moreover, the risk of developing diabetes in children due to sugar-containing medications cannot be overlooked. Furthermore, these practices contribute to the emergence of antibiotic resistance among children, with children as young as four years old found to harbor multidrug-resistant bacteria in their oral cavities [3]. The use of antibiotics in odontogenic infections should be limited to severe infections such as cellulitis, fasciitis, osteomyelitis, and deep neck-space infections, guided by the knowledge of the spectrum of the bacteria responsible for the infection, the virulence factors produced, and the host immune response [3,29]. 

In conclusion, more concrete and definitive guidelines are needed for managing orofacial bacterial infections and antibiotic prescribing in pediatric dental patients.

## Data Availability

Data supporting reported results can be found on Scopus, Web of Science, Google Scholar, and PubMed databases.
